# Which Factors Influence Functional Patients Improvements During Rehabilitation?

**DOI:** 10.5539/gjhs.v6n3p74

**Published:** 2014-02-20

**Authors:** Messina Gabriele, Rasimelli Lorena, Bonavita Chiara, Ceriale Emma, Quercioli Cecilia, Nante Nicola

**Affiliations:** 1 University of Siena, Department of Molecular and Developmental Medicine, Health Services Research Laboratory, Italy; 2 Rehabilitation Hospital Passignano sul Trasimeno, Local Health Unit 2 of Umbria Region, Perugia, Italy; 3 University of Siena, Post Graduate School in Public Health, Italy

**Keywords:** rehabilitation, orthopaedics, neurology, recovery of function

## Abstract

**Background:**

Rehabilitation in patients with disabilities is an important aspect of tertiary prevention. Severity of disability, evaluated by global measures of autonomy, is essential for functional outcome evaluation.

**Aim:**

To determine the effectiveness of a rehabilitation programme in terms of percentage functional improvement (PFI); to verify the role of gender, age and length of stay (LOS), by motor and cognitive domains, on PFI.

**Design:**

Longitudinal study.

**Setting:**

An intensive rehabilitation hospital.

**Population:**

305 inpatients.

**Methods:**

The disability has been investigated using the *Functional Independence Measure* (FIM). Percentage differences between discharge and admission were calculated for FIM score. *Wilcoxon matched pair test* for the six areas and the two domains of the FIM score were calculated. The effect of LOS, gender and age on PFI were studied with Robust regression.

**Results:**

Neurological and Orthopaedic patients had improvements on Motor and Cognitive domains. The greatest gains were in the Self Care, Sphintere Control, Transfer and Locomotion Areas (p=<0.001). LOS was associated (p<0.001) with PFI while age resulted borderline significant (p=0.049) in the cognitive domain in Neurological patients.

**Conclusion:**

The rehabilitation improved the overall conditions of neurological and orthopaedic patients. LOS emerged as the most important determinant in PFI.

## 1. Introduction

Rehabilitation to restore autonomy and social activity in patients with disabilities has become an increasingly important aspect of tertiary prevention due to the increase in chronic-degenerative diseases. Increasing health costs and decreasing financial resources call for optimization of rehabilitation and a need to find treatment strategies that improve patient condition. Quantification of the efficacy of rehabilitation programmes can help optimize resource allocation and use (Kehusmaa et al., 2010).

Rehabilitation involves many professions working in synergy; they include physicians, nurses, physiotherapists, occupational therapists, speech therapists, social workers, etc. How disabilities are cared for, with the management effects, especially in relation to the appropriateness, efficacy and equity of care, make it necessary to classify users in functional terms. Indeed, it has been shown that the cost of rehabilitation is determined by initial functional status, length of stay and the need for multidisciplinary care, rather than by diagnosis on admission (Harada, Sofaer, & Kominski, 1993; Rossnagel et al., 2005; Zorowitz, 2009).

Quantification of disabilities is possible with the *Functional Independence Measure* (FIM™), an international standard measurement of disability, whose main element is the FIM™ scale (Linacre et al., 1994; Stineman et al., 1997). Severity of disability, evaluated in the acute phase by global measures of autonomy such as the FIM scale, appear a powerful predictive variable for functional outcome (Adunsky et al., 1998; Linacre et al., 1994; Stineman et al., 1997; Tanaka et al., 2013). Moreover the FIM™ is able to assess both physical and cognitive functions. The applications of this scale ranges from summarizing information for patients with debility who received rehabilitation services, measuring the appropriateness and efficacy of rehabilitation in a single case, predicting needs and cost of care lending itself to clinical and administrative applications. (Galloway et al., 2013; Granger et al., 2011). Several studies have also investigated its application to the management of human resources in rehabilitation (Mueller et al. 2008; Mueller et al. 2010; Capolongo S. 2012).

Cumulative scores provide a quantitative index of disability making it possible to correlate scores with variables relevant for clinical and epidemiological purposes.

Along these lines, the aims of the present study are: i) to apply the FIM for measuring the functional status and its variation, in terms of mean percentage functional improvement (PFI), in patients which underwent rehabilitation program ii) to verify the role of gender, age and length of stay (LOS), by motor and cognitive domains, on PFI.

## 2. Materials and Methods

### 2.1 Setting

The study was conducted between January 2006 and June 2008 in a 20-bed ward of the Intensive Rehabilitation Hospital at Passignano on Trasimeno Lake, Perugia, Italy (Local Health Unit 2 on Umbria Region). The hospital is specialized in motor and neurological rehabilitation. Rehabilitation is aimed at two main categories of patients: neurological (mostly stroke hemiparesis patients) and orthopaedic (mostly hip and knee replacement patients). For the former, the rehabilitation programme consists of cognitive retraining exercises and, if necessary, rehabilitation of swallowing, neuromotor and daily activities, such as washing and dressing (motor activities with cognitive components). The occupational therapist, speech therapist and physiotherapist are involved in these activities, which take up about 3 hours per day, six days out of seven. Orthopaedic patients undergo about 2 hours per day of physiotherapy to recover full ranges of joint movements and proprioceptive exercises to gradually increase loading and force on the limb and reduce use of support for walking and negotiating stairs. All patients under normal admission conditions need 24-hour nursing care because they are not autonomous in any of the activities scored.

### 2.2 The Functional Independence Measure (FIM)

Disability on admission and at discharge was scored by FIM (Linacre et al. 1994; Stineman et al. 1997), which consists of 18 items assessing 6 areas of function, into two domains: Motor (13 items) and Cognitive (5 items). The Motor domain has 4 areas:

i) Self-care (SC) with 6 items: Eating, Grooming, Bathing, Dressing-upper body, Dressing-lower body, Toileting;
ii) Sphincter control (SPC) with 2 items: Bladder management, Bowel management;
iii) Transfers (T) with 3 items: Bed/chair/wheelchair, Toilet, Tub/shower;
iv) Locomotion (L), with 2 items: Walk/wheelchair, Stairs.

The Cognitive domains has 2 areas:

v) Communication (C) with 2 items: Comprehension, Expression;
vi) Social cognition (SOC) with 3 items: Social interaction, Problem solving, Memory.

Scores for each item range from 1 (complete dependence) to 7 (complete autonomy). The cumulative score of the different areas/items are standard indicators well known in the discipline of rehabilitation. The lowest score is 18 (indicating total dependence) and the highest is 126 (complete independence). If motor and cognitive items are considered separately, the former have a range of scores from 13 to 91 and the latter from 5 to 35. It never occurs that the score is uniform over all items/areas and therefore interpretation of the FIM makes it possible to define personalized care strategies, check objective limits of patients and apply personalized rehabilitation.

### 2.3 Selection of the Studied Population

The information obtained with the FIM forms by six experienced operators was analyzed in relation to data gathered from the hospital discharge forms in order to obtain insights into LOS, diagnosis and readmissions.

Inclusion criteria were: i) admission for rehabilitation; ii) cases judged to benefit from rehabilitation; iii) cases with neurological or orthopaedic Medical Diagnostic Classification (MDC) in which the main diagnosis had one of the following ICD9-CM codes: 2252, 2396, 33xx, 34xx, 3589, 41xx, 42.xx, 43xx, 4539, 5693, 5722, 71xx, 72xx, 78xx, 8088, 81409, 82xx, 85400, 90xx, 99671, V43xx, V49xx, V537, V549, V57xx.

Discharges of brief duration were regarded as a single admission, summing the days in hospital, in order to avoid double records. Such discharges occurred towards the end of the hospital period so that the team could assess the impact of the disability on the patient’s return home.

Exclusion criteria were: i) emergency transfer to another ward, preventing administration of the questionnaire at discharge; ii) incomplete answers to FIM; iii) patients in intensive care or otherwise unable to sustain 3 hours/day of rehabilitation.

After selection of the inclusion/exclusion criteria, the analysis was conducted on 305 out of a total of 879 cards. The cards were compiled within 3 days (72 hours) of admission and at discharge.

First we considered the total FIM score on admission and compared it with the score at discharge. Since the population was heterogeneous, we then considered the FIM score of each of the six areas (SC, SPC, T, L, C, SOC) and the subscores of the motor and cognitive domains, analyzing them on admission and at discharge in the two MDC categories: neurological (216 cases of stroke, hemiparesis with head injuries, multiple sclerosis, disabilities related to other diseases) and orthopaedic (89 cases of hip or knee replacement, cases of multiple trauma with leg or thigh amputation).

### 2.4 Statistical Analysis

Descriptive analysis of the data was performed; mean, median and interquartile values were calculated for the studied population using FIM score at the admission and discharge. Wilcoxon matched pair test for the six areas and the two domains was used to identify differences in the FIM score after treatment. Percentage functional improvement (PFI) between discharge and admission was calculated as following: difference between discharge FIM score (DS) and admission FIM score (AS), expressed in percentage:

PFI= (DS-AS)/DS*100. PFI makes possible to measure the improvements independently of the initial and final FIM score of the patients. It was globally calculated and then stratified for the motor and cognitive domains (outcome variables). PFI was adjusted with gender, age and LOS. Scatter plots using PFI and covariates were utilized to verify the possibility to use linear models. Robust regression, which aims to achieve almost the efficiency of ordinary least square regression in less ideal situations, such as when there are non normal errors, was adopted (Hamilton, 2012). Significance level was set p<0.05. Stata ® SE, version 12.1, StataCorp, College Station, Texas, USA software was used for the analysis.

## 3. Results

Our study sample was distributed as shown in Table 1. The largest age class was 70-79 years (42.3%). Women orthopaedic patients were almost two times more frequent than men. Mean ± (SD) LOS was 29.9 ± (23.9) days: 19.9 ± (12.3) days for orthopaedic and 34.0 ± (26.2) days for neurological patients.

**Table 1:** Distribution of orthopaedic and neurological patients by age class and gender

AGE	Orthopaedic		Neurological		TOTAL
	Men	Women	Men	Women	
**20 - 49**	6	2	12	12	32 (10.5%)
**50 - 59**	1	2	13	12	28 (9.2%)
**60 - 69**	3	8	26	17	54 (17.7%)
**70 - 79**	12	25	47	45	129 (42.3%)
**80 - 89**	6	24	13	19	62 (20.3%)
**TOTAL**	28 (9.2%)	61 (20.0%)	111 (36.4%)	105 (34.4%)	305

Table 2 shows global FIM scores at admission and discharge, divided in the motor and cognitive domains. Mean, median and interquartile range were calculated, for the global FIM score also subdivided by the motor and cognitive domains, both for orthopaedic and neurological patients.

**Table 2:** FIM scores on admission (entry) and at discharge (exit) for orthopaedic, neurological and all patients, by domain: mean (SD), median (interquartile range) and significance at difference between admission and discharge

Patients	Neurological					Orthopaedic					Global				
DOMAIN	ENTRY		EXIT			ENTRY		EXIT			ENTRY		EXIT		
	Mean (SD)	Median (Range)	Mean (SD)	Median (Range)	P	Mean (SD)	Median (Range)	Mean (SD)	Median (Range)	P	Mean (SD)	Median (Range)	Mean (SD)	Median (Range)	P
**Motor**	33.3 (18.4)	29 (18.3-43)	51.7 (20.4)	52 (36.3-68)	<0.001	51.2 (15.8)	51 (39-63.5)	67.6 (14.7)	69 (60.5-79)	<0.001	38.5 (19.4)	35 (21-52)	56.4 (20.2)	59 (41-72)	<0.001
**Cognitive**	25.2 (9.3)	28 (19-34)	27.7 (8.0)	30 (24.3-34)	<0.001	32.4 (4.6)	35 (31-35)	32.7 (4.2)	35 (32-35)	0.049	27.3 (8.8)	30 (23-35)	29.1 (7.4)	32 (26-35)	<0.001
**TOTAL**	58.5 (25.0)	55 (41-75)	79.4 (26.2)	81 (59.5-101)	<0.001	83.6 (18.5)	84 (70-98.5)	100.4 (17.4)	104 (92-114)	<0.001	65.8 (25.9)	65 (45.5-86)	85.5 (25.8)	90 (67-105)	<0.001

The Wilcoxon matched pair test identified significant improvements comparing the FIM scores at admission and discharge for orthopaedic and Neurological patients both at global and domain levels. The only exception was a borderline significance result in the cognitive domain (p=0.049) of the orthopaedic patients.

**Figure 1:**
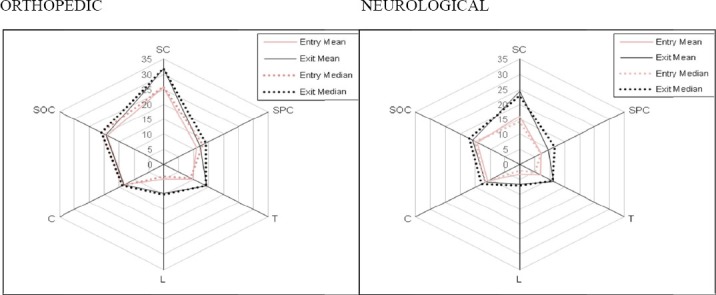
Means and medians at admission and discharge of hospital stay for the six FIM areas, distinguishing between orthopaedic and neurological cases

Figure 1 shows the means and medians at the admission and discharge of hospital stay for the six FIM areas, distinguishing between orthopaedic and neurological cases. Using the Wilcoxon matched pair test all patients areas highlighted statistically significant improvements (p=<0.001) except, in the Orthopaedic patients, the Communication (p=0.070) and Social cognition (p=0.057) areas, which both constitute the cognitive domain (p=0.049).

SC showed the greatest improvements both in Orthopaedic and Neurological areas followed by L and T areas.

Table 3 shows crude and adjusted, by LOS, gender and age, PFI, globally and by domains. Length of stay did improve PFI. Both Orthopaedic and Neurological patients benefit for LOS only on the motor domain. In particular in the adjusted model it increased, per every day of staying, 0.58 % (p<0.001) in orthopaedic patients and 0.32% (p<0.001) in neurological ones. The cognitive domain for Orthopaedic patients showed, an increase of 6.3% PFI in favour to female compared to male although borderline significant (p=0.053). Neurological patients, in the cognitive domain, showed a 0.05% increase (p=0.006) of PFI per every day of staying, while age resulted borderline significant (p=0.049) on PFI increasing 0.07% per every year of age.

**Table 3:** Crude and adjusted, by LOS, gender and age, PFI, globally and by domains

Crude* Percentage functional improvement in Motor, Cognitive domains, Global scale, Confidence intervals, P value										
Rehabilitation Type	Exposure variables	Motor Domain	95% CI	P	Cognitive Domain	95% CI	P	Global	95% CI	P
*Orthopedic*	Length stay^1^	0,563	0,374; 0,752	<0,001	-0,49	-0,312; 0,214	0,697	0,349	0,210; 0,490	<0,001
	gender^2^	-5,08	-10,63; 0,481	0,073	-2,59	-8,50; 3,314	0,365	-3,90	-7,68; -0,126	0,043
	age^3^	0,147	-0,046; 0,340	0,134	-0,034	-0,189; 0,121	0,65	0,077	0,057; 0,21	0,259
*Neurological*	Length stay^1^	0,32	0,218; 0,429	<0,001	0,053	0,024; 0,082	p<0,001	0,30	0,215; 0,380	<0,001
	gender^2^	3,17	-2,84; 9,19	0,300	-0,27	-1,82; 1,28	0,735	1,94	-3,04; 6,92	0,444
	age^3^	0,13	-0,098; 0,35	0,267	0,088	0,0222; 0,153	0,009	0,11	-0,070; 0,30	0,224

Adjusted** Percentage functional improvement in Motor, Cognitive domains, Global scale, Confidence intervals, P value										
Rehabilitation Type	Exposure variables	Motor Domain	95% CI	P	Cognitive Domain	95% CI	P	Global	95% CI	P
*Orthopedic*	Length stay^1^	0,576	0,39; 076	<0,001	-0,098	-0,336; 0,138	0,383	0,323	0,186; 0,46	<0,001
	gender^2^	-4,14	-9,24; 096	0,110	-6,294	-12,67; 0,082	0,053	-3,498	-7,23; 0,240	0,066
	age^3^	0,096	-0,080; 0,27	0,281	0,107	-0,26; 0,047	0,157	0,0328	-0,0964; 0,162	0,615
*Neurological*	Length stay^1^	0,32	0,212; 0,425	<0,001	0,05	0,014; 0,085	0,006	0,291	0,208; 0,380	<0,001
	gender^2^	2,68	-2,85; 8,21	0,340	-0,077	-1,92; 1,77	0,935	1,04	-3,29; 5,38	0,636
	age^3^	0,048	-0,159; 0,256	0,648	0,070	0,0004; 0,139	0,049	0,039	-0,123; 0,201	0,635

* Percentage variations for every single exposure variable

** Percentage variations adjusted for length of stay, gender and age

1 measured in days, 1 unit increase per day

2 male compared to female

3 measure in years; 1 unit increase per year

## 4. Discussion

In this study we examined the effectiveness of rehabilitation programme in terms of percentage functional improvement (PFI). We also verified the role of gender, age and LOS, by motor and cognitive domains, on PFI.

Rehabilitation in the hospitalized patients appear leading to an improvement in all subjects examined. The functional status measured with the FIM seems to improve for both orthopaedic and neurological patients. The orthopaedic patients had a better initial condition than neurological ones, this is confirmed by the fact that the former’s mean and medians admission scores in the six FIM areas (Figure 1) and domains (Table 2) were always higher.

Therefore, comparing the FIM scores at admission and discharge, we evidenced improvements for all patients and for both domains (motor and cognitive), with the exception of orthopaedic patients in the cognitive domain. This could be due to the fact that most orthopaedic patients did not have cognitive problems on admission, as neurological patients easily have. In fact cognitive impairment is a frequent complication of stroke in acute phase and is sometimes the severest and most evident symptom. Indeed, it is known, in patients with stroke, the importance of cognitive assessment in early and stabilization phases, in view of the interaction between different neuropsychological deficits and functional recovery (Pustokhanova & Morozova, 2013; Denti, Agosti, & Franceschini, 2008).

This may be because rehabilitation of stroke patients is intensive and caused this improvement in a brief time. This is also suggested by a meta-analysis (Kwakkel et al., 2004) and other studies (Feys et al., 2004; Sonoda et al., 2004) demonstrating that early intensive intervention produces an increase in FIM score at discharge and decreases the length of hospitalization.

The global PFI, in the adjusted models, is influenced by the independent variables: LOS, both in Orthopaedic and Neurological patients, changed slightly from its crude association; gender, on orthopaedic patients was border line significant (p=0.066) and its effect was not favourable for men respect to women. Length of stay has positive effect on PFI, in the motor domain, both in the orthopaedic and neurological patients, remaining similar at crude analysis. Orthopaedic patients, on the Cognitive domain, highlighted a borderline (p=0.053) reduction of -6.3% in male than in female.

This finding is partially similar to results obtained from another study which, analysing geriatric rehabilitation patients, evidenced that male ones were cognitively depressed; although women suffered more from pain and higher number of them presented with depressed mood (Arinzon et al., 2010).

We found that patients’ improvement take time to become evident, as indicated by small mean percentage gain during the rehabilitation process day by day. It also seems likely that hospitalization promotes overall improvement in neurological patients mainly due to the motor domain. The increment in FIM score as indicator of improvement of patient condition has been considered in prior studies. FIM score has also been proposed as a basis for reimbursement of rehabilitation centres (Harada, Sofaer, & Kominski, 1993; Stineman et al., 1998; Bottemiller et al., 2006) or as a factor correlated with LOS (Mahler et al. 2008; Grant, Goldsmith, and Anton 2014) adjusted according to the degree of patient disability (Bates & Stineman, 2000). Moreover, other studies highlighted and association among FIM and LOS (Cowen et al., 1995). We found similar evidence regarding the improvement of the patients’ conditions studying PFI, which measures percentage improvements depending the patients condition at admission and discharge, in relation to the effect of LOS, age, gender in the implementation of a rehabilitative care.

The present study is limited by the fact that the rehabilitation centre is specialized in stroke and orthopaedic patients. This means that our population was not fully representative of all categories of patient requiring rehabilitation such as cardiologic and burned ones, and the results therefore cannot be generalized. This problem could be avoided with a multicentre study covering hospitals with different specializations. Furthermore, the fact that we used rigid inclusion/exclusion criteria, enrolling 305 out of 879 subjects, reduced our population but made the data more reliable and less susceptible to variation.

## 5. Conclusions

In conclusion the results of this study confirm the importance of LOS in the improvements of the functional condition in patients who underwent rehabilitation although some differences emerged, among the Neurological and Orthopaedics groups. Some of these could have important repercussion on management programs also in terms of human and financial resources. This study had the merit to assess not only the FIM, but also its variation between admission and discharge, using mean percentage functional improvement (PFI).
